# Osteogenic differentiation of dental pulp stem cells under the influence of three different materials

**DOI:** 10.1186/s12903-015-0113-8

**Published:** 2015-10-28

**Authors:** Sumaiah A. Ajlan, Nahid Y. Ashri, Abdullah M. Aldahmash, May S. Alnbaheen

**Affiliations:** 1 Department of Periodontics and Community Dentistry, College of Dentistry, King Saud University, PO box: 65506, Riyadh, 11588 Saudi Arabia; 2 Stem cell unit, anatomy department, collage of medicine, King Saud University, Riyadh, Saudi Arabia; 3 Department of Endocrinology and Metabolism, Endocrine Research Laboratory (KMEB), Odense University Hospital & University of Southern Denmark, Odense, Denmark; 4 Dean of preparatory year, Saudi Electronic University, King Saud University, Riyadh, Saudi Arabia

**Keywords:** Dental pulp stem cells, Osteogenesis, MTA, EMD, PDG

## Abstract

**Background:**

Regeneration of periodontal tissues is a major goal of periodontal therapy. Dental pulp stem cells (DPSCs) show mesenchymal cell properties with the potential for dental tissue engineering. Enamel matrix derivative (EMD) and platelet-derived growth factor (PDGF) are examples of materials that act as signaling molecules to enhance periodontal regeneration. Mineral trioxide aggregate (MTA) has been proven to be biocompatible and appears to have some osteoconductive properties. The objective of this study was to evaluate the effects of EMD, MTA, and PDGF on DPSC osteogenic differentiation.

**Methods:**

Human DPSCs were cultured in medium containing EMD, MTA, or PDGF. Control groups were also established. Evaluation of the achieved osteogenesis was carried out by computer analysis of alkaline phosphatase (ALP)-stained chambers, and spectrophotometric analysis of alizarin red S-stained mineralized nodules.

**Results:**

EMD significantly increased the amounts of ALP expression and mineralization compared with all other groups (*P* < 0.05). Meanwhile, MTA gave variable results with slight increases in certain differentiation parameters, and PDGF showed no significant increase in the achieved differentiation.

**Conclusions:**

EMD showed a very strong osteogenic ability compared with PDGF and MTA, and the present results provide support for its use in periodontal regeneration.

## Background

The major goal of periodontal therapy is to regenerate tooth-supporting structures destroyed by periodontal disease [1]. Periodontal tissue engineering involves complex interactions between different cells and signaling molecules, as well as biological scaffolds [2].

In an attempt to mimic the original developmental events, the integrated use of precursor cell populations with specific biologic stimulants is under investigation [3, 4]. Stem cells represent primitive non-specialized cells with wide capabilities for differentiation and tissue regeneration. To date, mesenchymal stem cells have been successfully isolated from several body organs [5], including multiple tissues with dental origins [6–9]. Such dental tissue-derived stem cells were found to retain potent capacity for specific differentiation into dental tissue-forming cells [6, 10, 11]. Gronthos and colleagues successfully isolated human dental pulp stem cells (DPSCs), and proved both their multipotency and self-renewal capability [11, 12]. Further studies confirmed their findings [13, 14]. This multipotency, in addition to their relative accessibility, made DPSCs an appealing source of cells for application in regenerative medicine [15–18]. In fact, several papers have proved their superiority in different aspects, including osteogenic differentiation [19, 20], which supported their use for regeneration of craniofacial defects [21, 22], as well as alveolar bone defects [23, 24]. Additionally, the similar embryonic origins of dental pulp cells and periodontal cells [25] and their presence within protective layers of tooth structure have encouraged their use for periodontal tissue regeneration [26, 27].

Studies on tissue engineering have used biological mediators to selectively enhance the recruitment of cellular populations into periodontal wounds [28]. Enamel matrix derivative (EMD) is a protein harvested from developing porcine teeth that has been reported to induce cementum formation and periodontal regeneration [29]. At the cellular level, EMD was proven to have regulatory effects on multiple periodontal cell types [28, 30].

Platelet-derived growth factor (PDGF) is a very powerful regulatory factor that initiates nearly all wound healing events. The main function of PDGF is to stimulate cell replication (mitogenesis) of healing-capable stem cells and partially differentiated osteoprogenitor cells, which are part of the connective tissue–bone healing cellular make-up [31]. Significant increases in bone and cementum formation have been reported histologically [32]. At the cellular level, PDGF increased the number of collagen-synthesizing cells [33] and stimulated bone sialoprotein transcription [34].

Another material with the ability to induce regeneration is mineral trioxide aggregate (MTA). MTA is a mixture of dicalcium silicate, tricalcium silicate, tricalcium aluminate, gypsum, and tetracalcium aluminoferrite [35]. Torabinejad et al. [36] reported a favorable biologic performance of MTA when in direct contact with bone, through the deposition and formation of hydroxyl apatite on its surface. The material was also found to enhance cellular production of type I collagen, osteocalcin, alkaline phosphatase (ALP), bone sialoprotein, and osteopontin [37]. A systematic review on the histological responses of the periodontium to the material concluded that MTA promoted healing toward regeneration [38].

The above findings suggest similar clinical performances for the three materials with no previous attempts for direct comparisons. Accordingly, the purpose of the present study was to examine and compare the effects of EMD, PDGF, and MTA on the osteogenic differentiation of DPSCs.

## Results

### Cell isolation and characterization

**Fig. 1:**
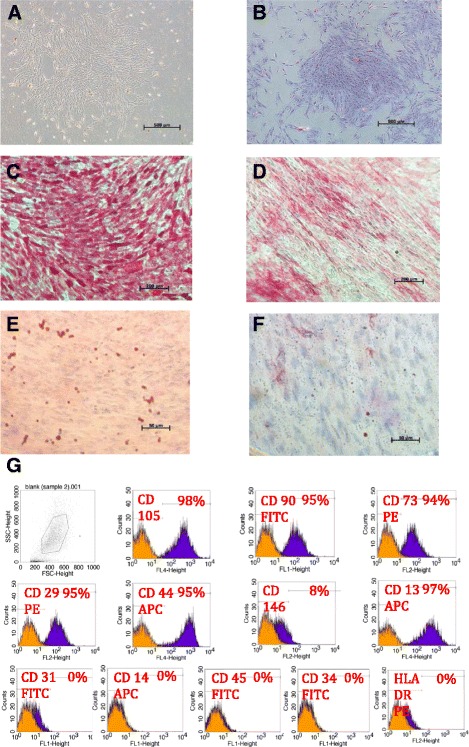
Inverted light microscopic images showing **a** dental pulp mesenchymal stem cells at primary culture, Magnification 5×. **b** Colony forming unit Fibroblast (CFU-F) magnification 5×, **c**, **d** Alkaline phosphatase staining for DPSCs 14 days after osteoinduction (**c**) versus negative control (**d**), magnification 10×, and Oil red O staining for DPSCs 14 days after adipogenic induction (**e**) versus negative control (**f**), magnification 40×. **g** FACS analysis results of a representative dental pulp cell line

Dental pulp stem cells in the primary cultures started to appear in 5–14 days and became attached to the plate surfaces (Fig. 1a). Cells from the second passage successfully formed multiple colonies, with around 50 cells per colony (Fig. 1b). Flow cytometry analyses confirmed positive expressions of stromal cell-associated markers, with negative expressions of hematopoietic and endothelial markers (Fig. 1g). Cells that underwent osteogenic induction showed increased ALP staining compared with negative control cells (Fig. 1c, d), while cells cultured in the adipogenic medium exhibited several oil red O-positive lipid granules (Fig. 1e, f).

### Material application

#### ALP staining

**Fig. 2:**
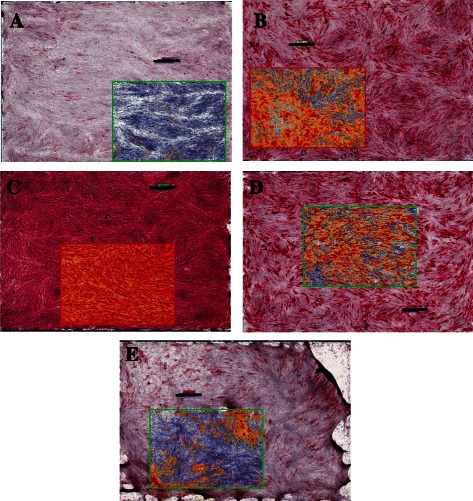
Scanoscope images for ALP stained different experimental groups of DPSCs. The internal image represents the evaluated field, which makes around 1/4^th^ of the image. Original magnification 1.4×. Scale bar 1 mm. **a** Negative control, **b** Reference control (OT), **c** EMD, **d** MTA, **e** PDGF

The samples showed different degrees of ALP staining (Fig. 2). One-way ANOVA revealed significant differences among the compared groups (*P* < 0.0001) (Table 1).

**Table 1:** Represents the alkaline phosphatase analysis results for all groups

	Material/Group	Average	Standard Deviation (SD)	Post hoc Tukey’s test for significance among groups
Percent Total Positive	Negative control	16.29	4.95	OT*
				EMD*
				MTA*
				PDGF*
	OT	72.92	9.24	Negative control*
				EMD*
				MTA*
				PDGF*
	EMD	95.59	4.69	Negative control*
				OT*
				MTA*
				PDGF*
	MTA	64.19	9.95	-ve control*
				OT*
				EMD*
				PDGF*
	PDGF	48.80	12.62	Negative control*
				OT*
				EMD*
				MTA*
Average Optical density	Negative control	0.18	0.01	OT*
				EMD*
				MTA*
				PDGF*
	OT	0.26	0.02	Negative control*
				EMD*
				MTA
				PDGF*
	EMD	0.35	0.03	Negative control*
				OT*
				MTA*
				PDGF*
	MTA	0.26	0.02	Negative control*
				OT
				EMD*
				PDGF
	PDGF	0.24	0.03	Negative control*
				OT*
				EMD*
				MTA
Histological Score	Negative control	20.633	7.034	OT*
				EMD*
				MTA*
				PDGF*
	OT	132.974	22.944	Negative control*
				EMD*
				MTA
				PDGF*
	EMD	221.992	23.818	Negative control*
				OT*
				MTA*
				PDGF*
	MTA	114.340	20.914	Negative control*
				OT
				EMD*
				PDGF*
	PDGF	82.330	28.254	Negative control*
				OT*
				EMD*
				MTA*

N.B.Intergroup comparison was statistically significant using ANOVA test, *P* < 0.0001

*Indicates statistical significance with *P* < 0.05

For all parameters examined, EMD was significantly higher than all other groups (*P* < 0.05). EMD revealed significantly higher percent total positive staining area, average optical density, and histological scores (95.6 ± 4.7 %, 0.35 ± 0.03, 221.99 ± 23.8) than MTA (64.19 %, 0.26 ± 0.02, 114.34 ± 20.90; *P* < 0.05) PDGF (48.8 % ± 12.62, 0.24 ± 0.02, 82.33 ± 28.3; *P* < 0.05) and reference control.

In contrast, MTA gave inconsistent findings, although it increased the ALP activity in a similar manner to the reference control when evaluated by the average optical density, the material resulted in reductions of the other parameters compared with the reference control, although those reductions were not always significant (*P* > 0.05).

With regard to PDGF, ALP expression generally revealed lower results compared with the reference control for the three parameters respectively, and these reductions were consistently significant (*P* < 0.05; Table 1).

#### Alizarin red S staining

**Fig. 3:**
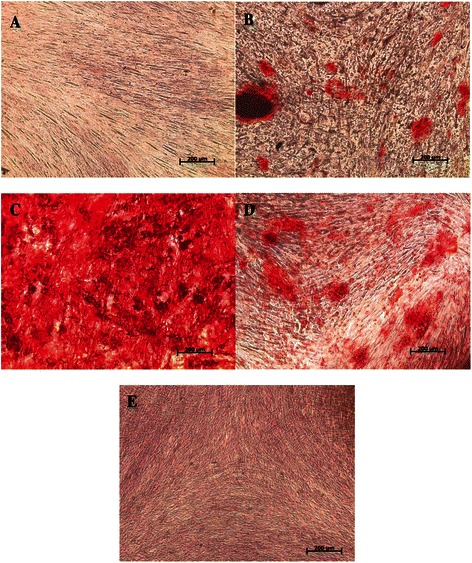
Inverted light microscopic image presenting Alizarin red S staining for different DPSCs experimental groups. Original magnification 10×. Scale bar 200 μm. **a** Negative control, **b** Reference control (OT), **c** EMD, **d** MTA, **e** PDGF

There were obvious differences in the amounts of mineralization among the groups (Fig. 3). One-way ANOVA revealed these differences to be significant (*P* < 0.0001) (Table 2).

**Table 2:** Represents the average absorbance rate for Alizarin red S stained chambers of all groups

Material/Group	Average	Standard Deviation (SD)	Post hoc Tukey’s test for significance among groups
Negative control	0.079	0.007	OT
			EMD*
			MTA*
			PDGF
OT	0.107	0.016	Negative control
			EMD*
			MTA
			PDGF
EMD	1.197	0.132	Negative control*
			OT*
			MTA*
			PDGF*
MTA	0.163	0.117	Negative control*
			OT
			EMD*
			PDGF*
PDGF	0.097	0.010	Negative control
			OT
			EMD*
			MTA*

*OT* reference control for osteoinduction, *EMD* Emdogain, *MTA* Mineral trioxide aggregate, *PDGF* Platelet derived growth factor-BB

N.B.Intergroup comparison was statistically significant using ANOVA test, *P* < 0.0001

*Indicates statistical significance with *P* < 0.05

The EMD group had a significantly increased amount of mineralized nodule formation compared with all other groups, giving a mean absorbance of 1.2 ± 0.13 (*P* < 0.05).

The MTA group significantly increased amount of mineralization (absorbance: 0.16 ± 0.12), relative to the negative control group (0.08 ± 0.01), and PDGF group (0.09 ± 0.01).

Although the mean absorbance of the PDGF group (0.09 ± 0.01) appeared to be slightly different than the other groups, these differences were statistically non-significant (*P* > 0.05; Table 2).

## Discussion

In this study, successful isolation of dental pulp cells was achieved through the application of enzymatic digestion with certain modifications to the protocol of Gronthos et al. [11]. The obtained cells underwent several investigations to evaluate their properties. According to the International Society for Cellular Therapy [39], the minimal criteria for defining multipotent mesenchymal stromal cells include: (1) adherence to plastic dishes; (2) multipotent differentiation potential; and (3) expressions of specific stromal surface markers (CD73, CD90, CD105) with lack of expressions of hematopoietic markers (CD45, CD34, CD14 and/or CD11b, CD19, CD79α) and the HLA-DR marker. The isolated cells in this study presented all of the above features.

Different material concentrations were evaluated, and the concentrations with the best differentiation were selected. These concentrations were 200 μg/ml for EMD, 5 ng/ml for PDGF, and 0.05 mg/ml for MTA. The same concentrations were previously used in other studies [34, 40, 41]. In this study, computer analysis for ALP activity and a semiquantitative evaluation technique for alizarin red S staining were selected, as these two techniques were reported to give results with relative sensitivity, and have been applied in previous studies [42, 43].

For EMD, the results revealed significant increases in ALP expression and abundant mineralization enhancement following its application. These findings are in accordance with several other studies evaluating the effects of this material on multiple cell lines [40, 44–48]. Duan et al. [44] found that EMD enhanced the osteogenic differentiation of induced pluripotent stem cell, as evidenced by increases in RUNX2 mRNA expression. Kémoun et al. [45, 46] evaluated the effects of EMD on follicular cells [45] and periodontal ligament stem cells [46]. In both studies, EMD was found to enhance ALP release and calcium deposition, in addition to the elevation of several mineralization markers. Another study by Guven et al. [47] found that Emdogain was the most effective material for enhancing both proliferation and odontogenic differentiation of human tooth germ stem cells through the evaluation of ALP activity, Von Kossa staining, and RT-PCR analyses for dentin sialophosphoprotein (DSPP), and immunostaining for collagen type I and DSPP. A study by Wang et al. [48] found that Emdogain enhanced the mineralization of DPSCs as well as their osteogenic/odontogenic marker expression. However, studies with contradictory findings are also available [49, 50]. It was reported that EMD might not have appreciable effects on osteoblastic differentiation in periodontal ligament cells [49] or rat bone marrow cells [50]. Although the exact control mechanism remains unclear, these effects were explained by differences in the degrees of cellular immaturity, i.e. the material was thought to enhance cellular proliferation of more immature cells, but differentiation of cells at later stages of maturity [51].

In the present study, MTA gave inconsistent findings. The material revealed mineralization enhancement in comparison with the reference control, reductions in certain ALP parameters (percent total positive staining area and histological score), and maintenance of other parameters (average optical density). Although Yasuda et al. [52] and Lee et al. [53] reported that MTA increased ALP production and/or mineralized nodule formation compared with control cells, both Koh et al. [54] and Nakayama et al. [55] reported similar ALP expression between MTA-treated cells and negative control cells. These inconsistencies suggest that further evaluation of the different parameters guiding and affecting the performance of this material is warranted.

With regard to PDGF in the present study, it was observed that ALP expression generally revealed lower results in comparison with the negative control group as well as all of the other material groups, and the differences were always significant. Regardless of the material’s action in proliferative enhancement, PDGF-BB appeared to have no additional benefit for osteogenic differentiation, according to the parameters evaluated in this study. Several other authors observed similar results [33, 56]. In fact, PDGF enhanced bone collagen degradation [33], and disrupted or inhibited bone matrix formation [56]. Nakashima et al. [57] found that PDGF increased DNA synthesis, while causing 40–65 % inhibition of ALP activity. Tanaka and Liang [58] reported that the material exerted no effect on cellular ALP activity or collagen synthesis. Yokose et al. [59] reported that PDGF-BB significantly reduced the ALP activity of DPSCs.

## Conclusions

Favorable cell-surface interactions with EMD were demonstrated, including ALP expression and abundant mineralization. EMD gave superior results compared with MTA and PDGF regarding osteogenic differentiation of DPSCs. The effects of MTA on osteogenesis of DPSCs were inconclusive and further studies are required. Moreover, our data on PDGF did not support its ability to induce osteogenic differentiation of DPSCs. However, PDGF did facilitate cell attachment and growth, suggesting a different mechanism of action that worth further investigation.

## Methods

### Isolation of stem cells

Human DPSCs were isolated and characterized by the authors in the Stem Cell Unit, King Saud University, Kingdom of Saudi Arabia (unpublished data). Teeth were collected from patients after they provided signed informed consent, according to a protocol approved by the institutional ethical committee (College of Dentistry Research Center-CDRC).

Briefly, the pulp contents of freshly extracted molar teeth were combined and subjected to 20–40 minutes of enzymatic digestion using collagenase type I (1 mg/ml) and dispase (5000 caseinolytic units). Subsequently, the cells were allowed to grow under regular cell culture conditions (37 °C, 5 % CO_2_), using Dulbecco’s modified Eagle’s medium (DMEM) supplemented with 20 % fetal bovine serum (FBS), 1 % penicillin-streptomycin (Pen-Strept), and 1 % non-essential amino acids (all purchased from Gibco-Invitrogen, USA).

### Characterization of stem cells

#### Colony forming unit-fibroblasts (CFU-F)

CFU-F were evaluated by culturing 2.5 × 10^3^ cells at the second passage in 6-cm culture dishes. At day 14, the cells were fixed with 1 % paraformaldehyde, stained with 0.5 % crystal violet, and subjected to microscopic evaluation using a phase-contrast inverted light microscope (Zeiss, Leica, Germany).

#### Flow cytometry

Fourth passage cells (1.5 × 10^6^) were washed with FACS buffer (1× phosphate-buffered saline, 5 % FBS, 0.1 % sodium azide), and diluted in 1.5 ml of phosphate-buffered saline. Next, PE-conjugated mouse anti-human CD146, CD73, CD29, and HLA-DR, FITC- conjugated mouse anti-human CD34, CD90, CD45, CD13, and CD31, and APC-conjugated mouse anti-human CD105, CD14, and CD44 antibodies were prepared in dark (all from BD Biosciences, USA, except for the monoclonal antibody against human CD105, which was purchased from R&D Systems, USA) and utilized. In each FACS tube, 100 μl of cells was mixed with 10 μl of the corresponding antibody, and incubated for 30 minutes in the dark at 4 °C. The expressions of cellular markers were assessed using a Becton Dickinson FACSCalibur Flow Cytometer (BD Biosciences, USA), and the resulting data were analyzed using Cell Quest Pro Software Version 3.3, BD bioscience, USA).

#### Osteogenic and adipogenic differentiation

Cells at the fourth passage were cultured on 6-well plates. At 60–70 % confluency, osteogenic differentiation was induced using osteoinduction medium prepared according to the protocol of Vishnubalaji et al. [60], and composed of DMEM supplemented with 10 % FBS, 1 % Pen-Strept, 50 μg/ml L-ascorbic acid (Wako Chemicals GmbH, Germany), 10 mM glycerol phosphate disodium salt (β-glycerophosphate), 10 nM dexamethasone, and 10 nM calcitriol (1α,25-dihydroxyvitamin D3) (Sigma, UK). Cells maintained in the regular culture medium served as controls. The resultant osteogenesis was evaluated after 14 days through cytochemical staining for ALP.

Adipogenic differentiation was also induced using standard adipogenic medium [60], composed of DMEM supplemented with 10 % FBS, 10 % horse serum, 1 % Pen-Strept, 100 nM dexamethasone, 0.45 mM isobutyl methyl xanthine, 3 μg/ml insulin (all purchased from Sigma, UK), and 1 μM rosiglitazone (BRL49653; Novo Nordisk, Denmark). The resultant differentiation was assessed at 14 days through the use of oil red O staining.

### Material application

**Fig. 4:**
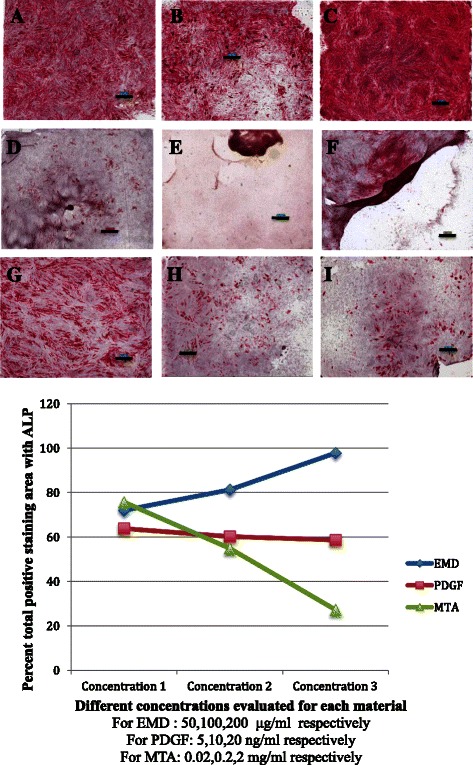
Scanoscope images for ALP stained different experimental groups of DPSCs. Original magnification 1.4×. Scale bar 1 mm. **a**, **b**, **c** EMD at concentrations of 50, 100, 200 μg/ml respectively. **d**, **e**, **f** PDGF at concentrations of 5, 10, 20 ng/ml respectively. **g**, **h**, **i** MTA at concentrations of 0.02, 0.2, 2.0 mg/ml respectively. **j** Percent total positive staining area for different concentrations of each examined material

Initially, a pilot study was carried out to evaluate three different concentrations for each material, and the concentrations yielding the highest amount of differentiation were selected for the comparisons (Fig. 4). Thereafter, cells at the fourth passage were cultured and divided into five groups as shown below.

1. **Negative Control:** Cells maintained in the regular cell culture medium for the entire experiment (DMEM with 20 % FBS, 1 % Pen-Strept, 1 % non-essential amino acids).
2. **Reference Control (OT):** Cells cultured in the osteoinduction medium, prepared according to the protocol of Vishnubalaji et al. [60].
3. **EMD Group:** Cells cultured in the osteoinduction medium supplemented with 200 μg/ml EMD (Straumann, USA).
4. **PDGF Group:** Cells cultured in the osteoinduction medium supplemented with 5 ng/ml PDGF-BB (Osteohealth, USA).
5. **MTA Group:** Cells cultured in the osteoinduction medium supplemented with 0.02 mg/ml MTA (Dentsply, USA).

The achieved differentiation was analyzed by evaluation of ALP expression through ALP staining and calcium ion deposition through alizarin red S staining.

#### ALP activity

Cells were plated on 8-chamber slides at the density of 0.02 × 10^6^ cells/chamber and allowed to attach and grow to 50 % confluency. Thereafter, the slides were divided into the above-mentioned five different groups and regular or osteogenic medium was applied accordingly. On day 5, the cells were fixed and stained for ALP with Naphthol-AS-TR-phosphate solution (Sigma, UK). Next, the chambers were evaluated under a high-resolution digital microscope where the whole stained chambers were scanned with a ScanScope slide scanner (Aperio Technologies Inc., USA) at 40× objective magnification. The digital images of six different chambers from each trial were viewed and analyzed using the viewing and image analysis tools of Aperio Image Scope software (Version 10.2.2.2352; Aperio Technologies Inc.). The whole experiment was repeated three times independently, giving a total of 18 chambers/group for analysis. The analysis output results were exported to Excel sheets, focusing mainly on the percent total positive staining area, average optical density, and histological score as the parameters for statistical analysis and comparison.

#### Alizarin red S staining

In the same manner, cells were cultured on 24-well plates, and the five different groups were established. Media were replaced twice per week with freshly-prepared regular or osteogenic media. On day 12, the cells were stained with 40 mM AR-S Alizarin Red (Sigma, UK), and subjected to spectrophotometric evaluation according to the protocol of Gregory et al. [61] using a microplate reader (Gen5™, version 1.10; BioTek Instruments Inc., USA) to measure the absorbance at 405 nM. The same protocol was repeated three times independently, giving nine different readings for each trial.

### Statistical analysis

Data was analyzed using SPSS statistical software (version 16.0; SPSS, USA). Descriptive statistics (mean and standard deviation) were used to describe the quantitative outcome variables. One-way analysis of variance (ANOVA) was used to compare the mean values of outcome variables across the categorical variables (groups), followed by a post-hoc Tukey test for pairwise comparisons. Values of *P* < 0.05 were considered to indicate statistical significance.

## Abbreviations

ALP: Alkaline phosphatase
AR-S: Alizarin red S stain
BRL: Rosiglitazone
°C: Degree celsius or degree centigrade
CD: Cluster of differentiation
CFU-F: Colony forming unit-fibroblast
DMEM: Dulbecco’s modified eagles medium (with high glucose, sodium pyrovate and L-glutamine)
DNA: Deoxy-ribionucleic acid
DPSCs: Dental pulp stem cells
EMD: Enamel matrix derivatives
FACS: Fluorescence-activated cell sorting (Flow cytometric analysis)
FBS: Fetal bovine serum
FITC: Fluorescence iso thioocyanide
Mg: Milligrams
ml: Milliliter
μl: Micro liters
mRNA: Messenger ribonucleic acid
MTA: Mineral trioxide aggregate
PBS: Phosphate buffered saline
Pen/Strept: Penicillin/Streptomycin
PDGF: Platelet derived growth factor
mRNA: Ribonucleic acid

## References

[1] Zander HA, Polson AM, Heijl LC (1976). Goals of periodontal therapy. J Periodontol.

[2] Benatti BB, Silverio KG, Casati MZ, Sallum EA, Nociti FH (2007). Physiological features of periodontal regeneration and approaches for periodontal tissue engineering utilizing periodontal ligament cells. J Biosci Bioeng.

[3] Monsarrat P, Vergnes JN, Nabet C, Sixou M, Snead ML, Planat-Benard V (2014). Concise review: mesenchymal stromal cells used for periodontal regeneration: a systematic review. Stem cells translational medicine.

[4] Yan XZ, Yang F, Jansen JA, de Vries RB, van den Beucken JJ (2015). Cell-Based Approaches in Periodontal Regeneration: A Systematic Review and Meta-Analysis of Periodontal Defect Models in Animal Experimental Work. Tissue Eng Part B Rev.

[5] Buzhor E, Leshansky L, Blumenthal J, Barash H, Warshawsky D, Mazor Y (2014). Cell-based therapy approaches: the hope for incurable diseases. Regen Med.

[6] Bansal R, Jain A (2015). Current overview on dental stem cells applications in regenerative dentistry. Journal of natural science, biology, and medicine.

[7] Bojic S, Volarevic V, Ljujic B, Stojkovic M (2014). Dental stem cells--characteristics and potential. Histol Histopathol.

[8] Huang GT, Gronthos S, Shi S (2009). Mesenchymal stem cells derived from dental tissues vs. those from other sources: their biology and role in regenerative medicine. J Dent Res.

[9] Saito MT, Silverio KG, Casati MZ, Sallum EA, Nociti FH (2015). Tooth-derived stem cells: Update and perspectives. World journal of stem cells.

[10] Eleuterio E, Trubiani O, Sulpizio M, Di Giuseppe F, Pierdomenico L, Marchisio M (2013). Proteome of human stem cells from periodontal ligament and dental pulp. PLoS One.

[11] Gronthos S, Mankani M, Brahim J, Robey PG, Shi S (2000). Postnatal human dental pulp stem cells (DPSCs) in vitro and in vivo. Proc Natl Acad Sci U S A.

[12] Gronthos S, Brahim J, Li W, Fisher LW, Cherman N, Boyde A (2002). Stem cell properties of human dental pulp stem cells. J Dent Res.

[13] Kraft DC, Bindslev DA, Melsen B, Abdallah BM, Kassem M, Klein-Nulend J (2010). Mechanosensitivity of dental pulp stem cells is related to their osteogenic maturity. Eur J Oral Sci.

[14] Pisciotta A, Carnevale G, Meloni S, Riccio M, De Biasi S, Gibellini L (2015). Human dental pulp stem cells (hDPSCs): isolation, enrichment and comparative differentiation of two sub-populations. BMC Dev Biol.

[15] Syed-Picard FN, Du Y, Lathrop KL, Mann MM, Funderburgh ML, Funderburgh JL (2015). Dental pulp stem cells: a new cellular resource for corneal stromal regeneration. Stem cells translational medicine.

[16] Kawashima N (2012). Characterisation of dental pulp stem cells: a new horizon for tissue regeneration?. Arch Oral Biol.

[17] Kabir R, Gupta M, Aggarwal A, Sharma D, Sarin A, Kola MZ (2014). Imperative role of dental pulp stem cells in regenerative therapies: a systematic review. Nigerian journal of surgery: official publication of the Nigerian Surgical Research Society.

[18] Spyridopoulos T, Lambropoulou M, Pagonopoulou O, Birbilis T, Tsaroucha AK, Kouzi-Koliakou K (2015). Regenerated Nerve Defects with a Nerve Conduit Containing Dental Pulp Stem Cells in Pigs: An Immunohistochemical and Electrophysiological Evaluation. J Reconstr Microsurg.

[19] Ito K, Yamada Y, Nakamura S, Ueda M (2011). Osteogenic potential of effective bone engineering using dental pulp stem cells, bone marrow stem cells, and periosteal cells for osseointegration of dental implants. Int J Oral Maxillofac Implants.

[20] Davies OG, Cooper PR, Shelton RM, Smith AJ, Scheven BA (2015). A comparison of the in vitro mineralisation and dentinogenic potential of mesenchymal stem cells derived from adipose tissue, bone marrow and dental pulp. J Bone Miner Metab.

[21] Yeon Kwon D, Seon Kwon J, Hun Park S, Hun Park J, Hee Jang S, Yun Yin X (2015). A computer-designed scaffold for bone regeneration within cranial defect using human dental pulp stem cells. Sci Rep.

[22] Annibali S, Bellavia D, Ottolenghi L, Cicconetti A, Cristalli MP, Quaranta R (2014). Micro-CT and PET analysis of bone regeneration induced by biodegradable scaffolds as carriers for dental pulp stem cells in a rat model of calvarial “critical size” defect: Preliminary data. J Biomed Mater Res B Appl Biomater.

[23] d’Aquino R, De Rosa A, Lanza V, Tirino V, Laino L, Graziano A (2009). Human mandible bone defect repair by the grafting of dental pulp stem/progenitor cells and collagen sponge biocomplexes. Eur Cell Mater.

[24] Liu HC ELL, Wang DS, Su F, Wu X, Shi ZP, Lv Y (2011). Reconstruction of alveolar bone defects using bone morphogenetic protein 2 mediated rabbit dental pulp stem cells seeded on nano-hydroxyapatite/collagen/poly(L-lactide). Tissue Eng Part A.

[25] Tziafas D, Kodonas K (2010). Differentiation potential of dental papilla, dental pulp, and apical papilla progenitor cells. J Endod.

[26] Aimetti M, Ferrarotti F, Cricenti L, Mariani GM, Romano F (2014). Autologous dental pulp stem cells in periodontal regeneration: a case report. Int J Periodontics Restorative Dent.

[27] Khorsand A, Eslaminejad MB, Arabsolghar M, Paknejad M, Ghaedi B, Rokn AR (2013). Autologous dental pulp stem cells in regeneration of defect created in canine periodontal tissue. J Oral Implantol.

[28] Venezia E, Goldstein M, Boyan BD, Schwartz Z (2004). The use of enamel matrix derivative in the treatment of periodontal defects: a literature review and meta-analysis. Critical Reviews of Oral Biology and Medicine.

[29] Hammarstrom L, Heijl L, Gestrelius S (1997). Periodontal regeneration in a buccal dehiscence model in monkeys after application of enamel matrix proteins. J Clin Periodontol.

[30] Gestrelius S, Andersson C, Lidstrom D, Hammarstrom L, Somerman M (1997). In vitro studies on periodontal ligament cells and enamel matrix derivative. J Clin Periodontol.

[31] Heldin CH, Westermark B (1999). Mechanism of action and in vivo role of platelet-derived growth factor. Physiol Rev.

[32] Lynch SE, de Castilla GR, Williams RC, Kiritsy CP, Howell TH, Reddy MS (1991). The effects of short-term application of a combination of platelet-derived and insulin-like growth factors on periodontal wound healing. J Periodontol.

[33] Canalis E, McCarthy TL, Centrella M (1989). Effects of platelet-derived growth factor on bone formation in vitro. J Cell Physiol.

[34] Mezawa M, Araki S, Takai H, Sasaki Y, Wang S, Li X (2009). Regulation of human bone sialoprotein gene transcription by platelet-derived growth factor-BB. Gene.

[35] Camilleri J, Pitt Ford TR (2006). Mineral trioxide aggregate: a review of the constituents and biological properties of the material. Int Endod J.

[36] Torabinejad M, Hong CU, Pitt Ford TR, Kaiyawasam SP (1995). Tissue reaction to implanted super-EBA and mineral trioxide aggregate in the mandible of guinea pigs: a preliminary report. J Endod.

[37] Chen CL, Huang TH, Ding SJ, Shie MY, Kao CT (2009). Comparison of calcium and silicate cement and mineral trioxide aggregate biologic effects and bone markers expression in MG63 cells. J Endod.

[38] Katsamakis S, Slot DE, Van der Sluis LW, Van der Weijden F (2013). Histological responses of the periodontium to MTA: a systematic review. J Clin Periodontol.

[39] Dominici M, Le Blanc K, Mueller I, Slaper-Cortenbach I, Marini F, Krause D (2006). Minimal criteria for defining multipotent mesenchymal stromal cells. The International Society for Cellular Therapy position statement. Cytotherapy.

[40] Song ZC, Shu R, Zhang XL (2010). Cellular responses and expression profiling of human bone marrow stromal cells stimulated with enamel matrix proteins in vitro. Cell Prolif.

[41] Zhao X, He W, Song Z, Tong Z, Li S, Ni L (2012). Mineral trioxide aggregate promotes odontoblastic differentiation via mitogen-activated protein kinase pathway in human dental pulp stem cells. Mol Biol Rep.

[42] Krause U, Seckinger A, Gregory CA (2011). Assays of osteogenic differentiation by cultured human mesenchymal stem cells. Methods Mol Biol.

[43] Bruedigam C, Driel M, Koedam M, Peppel J, van der Eerden BC, Eijken M, van Leeuwen JP: Basic techniques in human mesenchymal stem cell cultures: differentiation into osteogenic and adipogenic lineages, genetic perturbations, and phenotypic analyses. Current protocols in stem cell biology. 2011;Chapter 1:Unit1H 3. 10.1002/9780470151808.sc01h03s17 21633940

[44] Duan X, Tu Q, Zhang J, Ye J, Sommer C, Mostoslavsky G (2011). Application of induced pluripotent stem (iPS) cells in periodontal tissue regeneration. J Cell Physiol.

[45] Kemoun P, Laurencin-Dalicieux S, Rue J, Farges JC, Gennero I, Conte-Auriol F (2007). Human dental follicle cells acquire cementoblast features under stimulation by BMP-2/-7 and enamel matrix derivatives (EMD) in vitro. Cell Tissue Res.

[46] Kemoun P, Gronthos S, Snead ML, Rue J, Courtois B, Vaysse F (2011). The role of cell surface markers and enamel matrix derivatives on human periodontal ligament mesenchymal progenitor responses in vitro. Biomaterials.

[47] Guven EP, Yalvac ME, Sahin F, Yazici MM, Rizvanov AA, Bayirli G (2011). Effect of dental materials calcium hydroxide-containing cement, mineral trioxide aggregate, and enamel matrix derivative on proliferation and differentiation of human tooth germ stem cells. J Endod.

[48] Wang Y, Zhao Y, Ge L (2014). Effects of the enamel matrix derivative on the proliferation and odontogenic differentiation of human dental pulp cells. J Dent.

[49] Okubo K, Kobayashi M, Takiguchi T, Takada T, Ohazama A, Okamatsu Y (2003). Participation of endogenous IGF-I and TGF-beta 1 with enamel matrix derivative-stimulated cell growth in human periodontal ligament cells. J Periodontal Res.

[50] van den Dolder J, Vloon AP, Jansen JA (2006). The effect of Emdogain on the growth and differentiation of rat bone marrow cells. J Periodontal Res.

[51] Yoneda S, Itoh D, Kuroda S, Kondo H, Umezawa A, Ohya K (2003). The effects of enamel matrix derivative (EMD) on osteoblastic cells in culture and bone regeneration in a rat skull defect. J Periodontal Res.

[52] Yasuda Y, Ogawa M, Arakawa T, Kadowaki T, Saito T (2008). The effect of mineral trioxide aggregate on the mineralization ability of rat dental pulp cells: an in vitro study. J Endod.

[53] Lee SK, Lee SK, Lee SI, Park JH, Jang JH, Kim HW (2010). Effect of calcium phosphate cements on growth and odontoblastic differentiation in human dental pulp cells. J Endod.

[54] Koh ET, Torabinejad M, Pitt Ford TR, Brady K, McDonald F (1997). Mineral trioxide aggregate stimulates a biological response in human osteoblasts. J Biomed Mater Res.

[55] Nakayama A, Ogiso B, Tanabe N, Takeichi O, Matsuzaka K, Inoue T (2005). Behaviour of bone marrow osteoblast-like cells on mineral trioxide aggregate: morphology and expression of type I collagen and bone-related protein mRNAs. Int Endod J.

[56] Hock JM, Canalis E (1994). Platelet-derived growth factor enhances bone cell replication, but not differentiated function of osteoblasts. Endocrinology.

[57] Nakashima M, Nagasawa H, Yamada Y, Reddi AH (1994). Regulatory role of transforming growth factor-beta, bone morphogenetic protein-2, and protein-4 on gene expression of extracellular matrix proteins and differentiation of dental pulp cells. Dev Biol.

[58] Tanaka H, Liang CT (1995). Effect of platelet-derived growth factor on DNA synthesis and gene expression in bone marrow stromal cells derived from adult and old rats. J Cell Physiol.

[59] Yokose S, Kadokura H, Tajima N, Hasegawa A, Sakagami H, Fujieda K (2004). Platelet-derived growth factor exerts disparate effects on odontoblast differentiation depending on the dimers in rat dental pulp cells. Cell Tissue Res.

[60] Vishnubalaji R, Al-Nbaheen M, Kadalmani B, Aldahmash A, Ramesh T (2012). Comparative investigation of the differentiation capability of bone-marrow- and adipose-derived mesenchymal stem cells by qualitative and quantitative analysis. Cell Tissue Res.

[61] Gregory CA, Gunn WG, Peister A, Prockop DJ (2004). An Alizarin red-based assay of mineralization by adherent cells in culture: comparison with cetylpyridinium chloride extraction. Anal Biochem.

