# Erratum to: Osteogenic differentiation of dental pulp stem cells under the influence of three different materials

**DOI:** 10.1186/s12903-016-0193-0

**Published:** 2016-03-22

**Authors:** Sumaiah A. Ajlan, Nahid Y. Ashri, Abdullah M. Aldahmash, May S. Alnbaheen

**Affiliations:** Department of Periodontics and Community Dentistry, College of Dentistry, King Saud University, PO box: 65506, Riyadh, 11588 Saudi Arabia; Stem Cell Unit, Anatomy Department, Collage of Medicine, King Saud University, Riyadh, Saudi Arabia; Department of Endocrinology and Metabolism, Endocrine Research Laboratory (KMEB), Odense University Hospital & University of Southern Denmark, Odense, Denmark; Dean of Preparatory Year, Saudi Electronic University, King Saud University, Riyadh, Saudi Arabia

After publication of this work [1], the authors noticed that Figs. 1 and 4 are duplicated. The original version of this article was corrected. The publisher apologizes for any inconvenience caused.

**Fig. 1:**
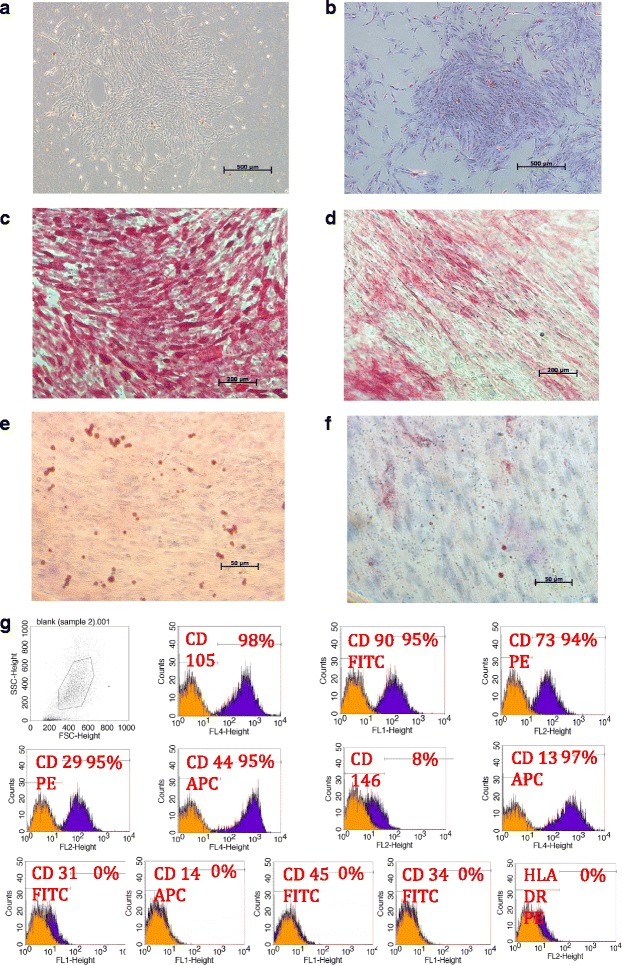
Inverted light microscopic images showing (**a**) dental pulp mesenchymal stem cells at primary culture, Magnification 5×. **b** Colony forming unit Fibroblast (CFU-F) magnification 5×, Alkaline phosphatase staining for DPSCs 14 days after osteoinduction (**c**) versus negative control (**d**), magnification 10×, and Oil red O staining for DPSCs 14 days after adipogenic induction (**e**) versus negative control (**f**), magnification 40×. (**g**) FACS analysis results of a representative dental pulp cell line

The correct Fig. 1 is given below:
